# In Silico Predicted Antifungal Peptides: In Vitro and In Vivo Anti-*Candida* Activity

**DOI:** 10.3390/jof7060439

**Published:** 2021-05-31

**Authors:** Tecla Ciociola, Walter Magliani, Tiziano De Simone, Thelma A. Pertinhez, Stefania Conti, Giorgio Cozza, Oriano Marin, Laura Giovati

**Affiliations:** 1 Department of Medicine and Surgery, University of Parma, 43126 Parma, Italy; tecla.ciociola@unipr.it (T.C.); walter.magliani@unipr.it (W.M.); tiziano.desimone@unipr.it (T.D.S.); thelma.pertinhez@unipr.it (T.A.P.); laura.giovati@unipr.it (L.G.); 2 Department of Molecular Medicine, University of Padua, 35131 Padua, Italy; giorgio.cozza@unipd.it; 3 CRIBI Biotechnology Center, University of Padua, 35131 Padua, Italy; oriano.marin@unipd.it; 4 Department of Biomedical Sciences, University of Padua, 35131 Padua, Italy

**Keywords:** antifungal peptides, *Candida albicans*, circular dichroism spectroscopy, *Galleria mellonella* model, in silico analysis, structure–activity relationship

## Abstract

It has been previously demonstrated that synthetic antibody-derived peptides could exert a significant activity in vitro, ex vivo, and/or in vivo against microorganisms and viruses, as well as immunomodulatory effects through the activation of immune cells. Based on the sequence of previously described antibody-derived peptides with recognized antifungal activity, an in silico analysis was conducted to identify novel antifungal candidates. The present study analyzed the candidacidal and structural properties of in silico designed peptides (ISDPs) derived by amino acid substitutions of the parent peptide KKVTMTCSAS. ISDPs proved to be more active in vitro than the parent peptide and all proved to be therapeutic in *Galleria mellonella* candidal infection, without showing toxic effects on mammalian cells. ISDPs were studied by circular dichroism spectroscopy, demonstrating different structural organization. These results allowed to validate a consensus sequence for the parent peptide KKVTMTCSAS that may be useful in the development of novel antimicrobial molecules.

## 1. Introduction

The development of antimicrobial drugs in the middle of the last century greatly improved the prognosis of infectious diseases, thus increasing life expectancy. Nonetheless, even today, infectious diseases, with particular reference to those caused by new and re-emerging etiologic agents, represent a common cause of death in different areas of the world [1].

Opportunistic fungal infections pose a special threat to particular at-risk populations, such as severely immunocompromised people, transplanted individuals, and oncologic patients. Among these, infections due to *Candida* spp. are the most common worldwide. Drugs to treat invasive fungal infections are limited to only a few approved classes and, despite the available treatments, mortality rates remain unacceptably high. Further problems are due to the increasing spread of resistance phenomena, although to a lesser extent compared with antibacterial drugs [2].

While significant efforts are ongoing in identifying novel antifungal compounds and classes, and optimizing the agents within the present antifungal arsenal, new strategies have also been approached to drug development, including the screening of approved drugs for drug repurposing [3,4,5]. Recent reviews described antifungal agents currently in various stages of clinical development [6,7,8,9].

Among the potential candidate drugs, a great number of antimicrobial peptides (AMPs) from different sources have been studied [10,11,12,13,14]. Thanks to their features, AMPs are attractive molecules for translational application, and dozens of them are currently being evaluated in clinical trials, although only a few as antifungals [15,16].

Moreover, new methods such as template-based, docking simulations, and other sequence-based methods allow for novel in silico prediction of antifungal peptides [17,18].

In this work, based on the sequence of previously described antibody-derived peptides with recognized antifungal activity, an in silico analysis was conducted aimed at the identification of novel antifungal peptides. The selected candidates proved to be more active in vitro than the parent peptide against a reference *Candida albicans* strain, without showing toxic effects on mammalian cells. All of them also exhibited a therapeutic effect in vivo in *Galleria mellonella* candidal infection. These results allow to validate a consensus sequence that could be useful to obtain optimized molecules from a recognized antimicrobial peptide.

## 2. Materials and Methods

### 2.1. In Silico Analysis

Computational analysis was performed starting from three previously described peptides endowed with anti-*Candida* activity. In particular, peptides K10S (KKVTMTCSAS) [19], D5A (TCRVAHRGLTF) [20], and N1A (AQVSLTCLVK) [21] were selected to determine the correspondences between residues of the three sequences. For this purpose, we exploited MOE’s sequence alignment tool, a modified version of the alignment methodology originally introduced into molecular biology by Needleman (Molecular Operating Environment, MOE, 2020.09, Chemical Computing Group ULC, Montreal, QC, Canada, 2020). The alignment was computed through a function based on residue similarity score (obtained from applying BLOSUM 40 substitution matrix) and gap penalties. Starting from the amino acidic sequence of K10S peptide, random peptides were generated through sample sequence methodology and analyzed through the mutational analysis tool.

### 2.2. Peptide Synthesis

K10S parent peptide and in silico designed peptides (ISDPs) derived from K10S by amino acidic substitutions were synthesized using the fluoren-9-ylmethoxycarbonyl (Fmoc) solid-phase synthesis chemistry, purified by HPLC, and analyzed by mass spectroscopy at CRIBI-Peptide Facility (University of Padua, Padua, Italy), as previously described [21]. A stock solution (20 mg/mL) of peptides was prepared in dimethyl sulfoxide (DMSO) and stored at 4 °C. Dilutions were made for evaluation of biological activities. Controls (without peptides) always contained DMSO at proper concentrations (maximum 0.5%).

### 2.3. Evaluation of the In Vitro Candidacidal Activity of ISDPs

The candidacidal activity of ISDPs was evaluated by conventional colony forming unit (CFU) assays, as previously described [22]. ISDPs were tested at serial dilutions to determine the half maximal effective concentration (EC_50_) values. Briefly, approximately 500 germinating *C. albicans* SC5314 cells were suspended in 100 µL of distilled water in the presence or absence (control growth) of ISDPs. After incubation at 37 °C for 6 h, cell suspensions were plated on Sabouraud dextrose agar. CFUs were enumerated after 48–72 h of incubation at 30 °C, and candidacidal activity was determined as a percentage of CFU reduction. Each assay was carried out in triplicate and at least two independent experiments were performed for each condition. EC_50_ was calculated by nonlinear regression analysis using GraphPad Prism 5 software. Afterwards, the kinetics of ISDPs killing activity, at their 2× EC_50_ value concentration, was determined by CFU assays up to 6 h. Samples were collected for CFU determination after 5, 10, 15, 20, 30, 60, 120, 240, and 360 min incubation.

### 2.4. Evaluation of the Hemolytic and Cytotoxic Effects of ISDPs

Hemolytic and cytotoxic effects were determined as previously described [22]. In particular, ISDPs (final concentrations of 25, 50, and 100 µM) were tested for their hemolytic activities against human red blood cells (hRBCs) (group 0 Rh+). After 30 and 120 min incubation at 37 °C, the release of hemoglobin was monitored by measuring the absorbance of the supernatant at 540 nm. Controls for zero hemolysis (blank) and 100% hemolysis consisted of hRBCs suspended in PBS and Triton 1%, respectively.

MTT assay, based on the ability of metabolically active cells to convert the yellow water-soluble tetrazolium salt into formazan crystals, was used to evaluate peptide cytotoxicity against monkey kidney epithelial cells (LLC-MK2). LLC-MK2 cells were treated with ISDPs (50, 100, and 200 µM) for 24 h. Cells in medium without peptide served as control. After this period, cells were incubated with MTT (5 mg/mL, 10 μL/well) in serum-free medium for 2 h at 37 °C, the medium was removed, and the crystal formazan dye was solubilized by adding isopropanol with 5% HCl 1 M (100 μL). Absorbance was measured at 540 nm.

### 2.5. Circular Dichroism (CD) Spectroscopy

Circular dichroism (CD) experiments were carried out using a Jasco 715 spectropolarimeter (JASCO International Co. Ltd., Tokyo, Japan), coupled to a Peltier PTC-348WI system for temperature control. Far-UV spectra were recorded at 20 °C in the range 250–190 nm, 0.5 nm wavelength steps, 50 nm/min scanning speed, 1.0 nm bandwidth, and four accumulations, using a 1 mm path length quartz cuvette. A starting aqueous solution (1 mM) of the parent K10S peptide and ISDPs was prepared and stored at 4 °C. For CD experiments, samples were diluted to a final concentration of 100 µM and analyzed immediately or 7 days and 24 months later. Following baseline correction, the observed ellipticity θ (millidegrees) was converted to molar mean residue ellipticity [θ] (deg cm^2^ dmol^−1^).

### 2.6. Evaluation of Apoptosis Induction and Reactive Oxygen Species (ROS) Production in C. albicans after Treatment with ISDPs

Induction of apoptosis after treatment with ISDPs was evaluated as previously described [21]. Yeast cells were suspended in water (5 × 10^5^ cells/mL), in absence (control) or presence of ISDPs, at their 2× EC_50_ value, for 30 min. For the evaluation of the apoptotic profile, the Muse Annexin V & Dead Cell Assay reagent (Merck Millipore, Merck KGaA, Darmstadt, Germany) was used. Data were acquired by the Muse Cell Analyzer (Merck Millipore) according to the manufacturer’s instructions. At least two independent experiments were performed for each condition. Data are reported as the mean ± standard deviation. Differences between ISDPs-treated groups and control or parent-treated group were assessed by unpaired two-tailed Student’s *t*-test. A value of *p* < 0.05 was considered significant.

ISDP-induced ROS production in *C. albicans* SC5314 cells was evaluated as previously described [19]. Briefly, yeast cells (2 × 10^7^ cells/mL) were suspended in 110 µL water in the presence or absence of 25 mM ascorbic acid and incubated for 30 min. Then, ISDPs were added at concentration 30× their EC_50_ value in a final volume of 220 µL. As positive control, yeast cells were incubated in presence of 20 µg/mL caspofungin. After 30 min incubation at 37 °C, cells were centrifuged and resuspended in 220 µL PBS pH 7.4 with 10 µg/mL 2′,7′-dichlorofluorescin diacetate (DCFH-DA). Then, 100 µL of suspensions were transferred in 96-well microplates for fluorescence (PerkinElmer™, Waltham, MA, USA) and incubated for 4 h at 37 °C. Fluorescent signal due to DCFH (derived by oxidation of DCFH-DA) was measured at time 0 up to 4 h on an EnSpire plate reader (PerkinElmer™) at excitation and emission wavelength of 485 and 540 nm, respectively. Each assay was carried out in duplicate, and at least two independent experiments were performed for each condition.

### 2.7. Fluorescence Microscopy Studies

The potential role of ISDPs in membrane permeabilization in living *C. albicans* SC5314 cells was studied by fluorescence microscopy using a Nikon Eclipse 80i optical microscope, equipped with a Nikon Digital Sight DS-2Mv camera, and images were acquired with NIS Elements F control software (Nikon Co., Tokyo, Japan). Yeast cells grown in yeast extract, peptone, and dextrose broth overnight at 30 °C with shaking (100 rpm) were washed once with water and 4 × 10^7^ cells/mL were loaded with 500 μM Lucifer Yellow (LY) and 1.5 μM propidium iodide (PI). LY is a fluorescent molecule used as a quantitative marker of the cell membrane permeabilization [23], while PI is a non-vital nuclear stain commonly used for identifying dead cells. Yeast cell suspensions (10 μL) were seeded on Polysine Adhesion Slides (Thermo Scientific™, Thermo Fisher Scientific Inc., Waltham, MA, USA) and, after 10 min, ISDPs were added (final concentrations in the range 90–97 μM). Images were taken up to 30 min at a 40× magnification.

### 2.8. Evaluation of In Vivo Therapeutic Activity of ISDPs in Galleria mellonella

In vivo potential therapeutic effects of ISDPs were studied in *G. mellonella* larvae injected with a lethal dose of *C. albicans* SC5314 cells, as previously described [24]. Thirty minutes after *Candida* infection (5 × 10^5^ cells/larva in 10 µL of saline), larvae were injected via the last right pro-leg with ISDPs (13 µmol/kg) or saline (control) (16 larvae/group). Further control groups consisted of larvae untouched or inoculated with only 10 µL of saline solution. Larvae were then transferred into clean Petri dishes, incubated at 37 °C in the dark for 9 days, and scored daily for survival. Survival curves of ISDP-treated and control animals were compared by the Mantel–Cox log-rank test. A value of *p* < 0.05 was considered significant.

## 3. Results

### 3.1. In Silico Peptide Analysis and ISDPs’ Selection

The sequence alignment obtained for the three selected antimicrobial peptides (K10S, D5A and N1A, Table 1) suggests a pairwise percentage identity (PPI) of 10.0 for K10S versus D5A, 30.0 for K10S versus N1A, and 20.0 for D5A versus N1A. Notably, despite the low PPI, a slight trend in the amino acid sequences of the three peptides can be identified. Indeed, all three peptides present two hydrophobic residues, one of which is represented by a conserved valine, spaced by four (D5A and N1A) or five (K10S) amino acids. Based on this rationale, we focused on the K10S peptide in order to identify further active derivatives and, consequently, a possible consensus sequence that could be decisive for their antifungal activity. For this purpose, we have generated a small database of K10S derivatives characterized by substitution with basic, hydrophobic, or Ser/Thr residues. In particular, we have substituted the two Lys at the N-terminus with two Arg (R10S-RR), the Ala residue at C-terminus with an Ile (K10S-I), and inverted Thr with Ser, and vice versa in the two triplets T-X-T (becoming S-X-S, K10S-SS) and S-X-S (becoming T-X-T, K10T-TT).

In Table 2, the sequences and characteristics of the parent peptide and the selected ISDPs are reported.

### 3.2. In Vitro Candidacidal Activity of ISDPs

All ISDPs showed an increased candidacidal activity against *C. albicans* SC5314, in comparison with the parent peptide K10S, with EC_50_ values ranging between 0.159 and 0.260 µM (Table 3). The highest activity was observed with Thr/Ser and Ser/Thr substitutions.

**Figure 1:**
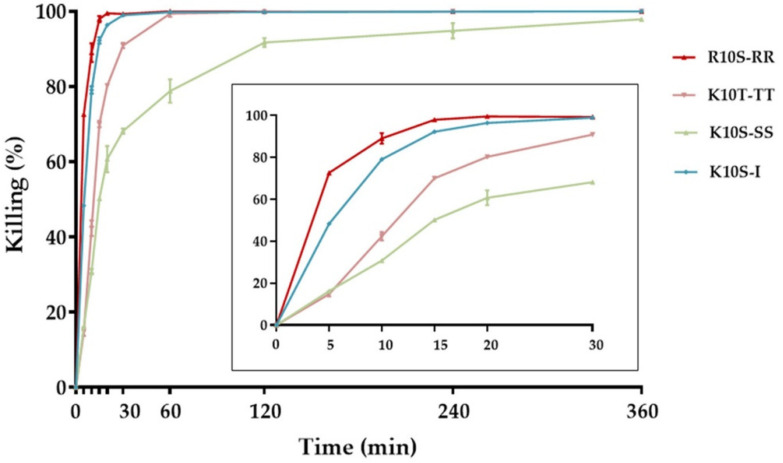
Time kinetics of in vitro activity of in silico designed peptides (ISDPs) against *Candida albicans* SC5314. Peptide concentrations were equal to their 2× EC_50_ values. The activity is expressed as percental killing; reported data represent mean values ± standard deviation. The inset shows in detail the killing kinetics in the first 30 min.

The rates of *C. albicans* killing of ISDPs over time are reported in Figure 1. Candidacidal activity of all tested peptides was very fast. After 30 min, in fact, percental killing ranged from 68% (K10S-SS) to nearly 100% (R-10S-RR and K10S-I). All ISDPs demonstrated a more rapid candidacidal effect in comparison with the parent K10S peptide, whose percental killing at 30 min was 36.93% [19].

### 3.3. Hemolytic and Cytotoxic Effects of ISDPs

In comparison with negative (PBS, 0% hemolysis) and positive controls (Triton 1%, 100% hemolysis), ISDPs (25, 50, and 100 µM) showed a negligible and concentration-independent hemolytic activity on human red blood cells. In fact, after 30 min incubation in the presence of the investigated peptides, lysed erythrocytes were always <1.4%, with the only exception of K10S-SS 50 µM (hemolysis 2.38%). After 120 min incubation, hemolysis was always <2%, with the only exception of K10S-SS 50 µM (hemolysis 2.01%).

Moreover, none of the investigated peptides showed a significant cytotoxicity against LLC-MK2 cells, as assessed by the MTT assay. After 24 h incubation with peptides, at all concentrations tested, mean absorbance values were higher than the ones of the untreated cells, with the only exception of K10S-SS 200 µM (in this case, cell viability was 92.5 ± 0.11% vs. 100% of untreated control cells).

### 3.4. ISDPs Conformational State

**Figure 2:**
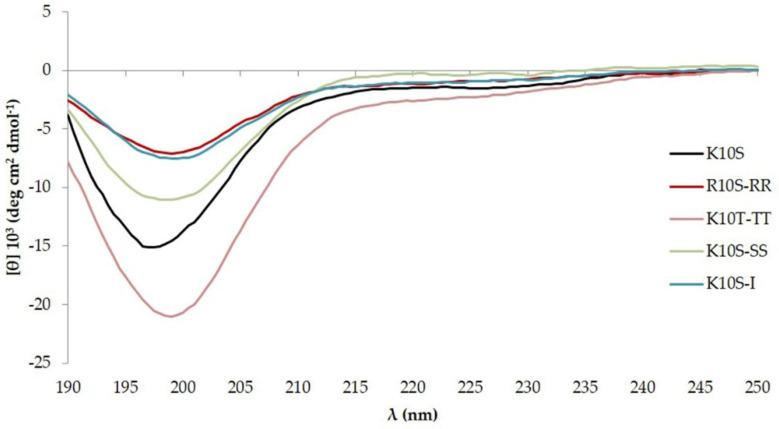
Far UV-CD spectra at 20 °C of 100 μM K10S parent peptide and ISDPs 7 days after preparation of the stock aqueous solution (1 mM).

**Figure 3:**
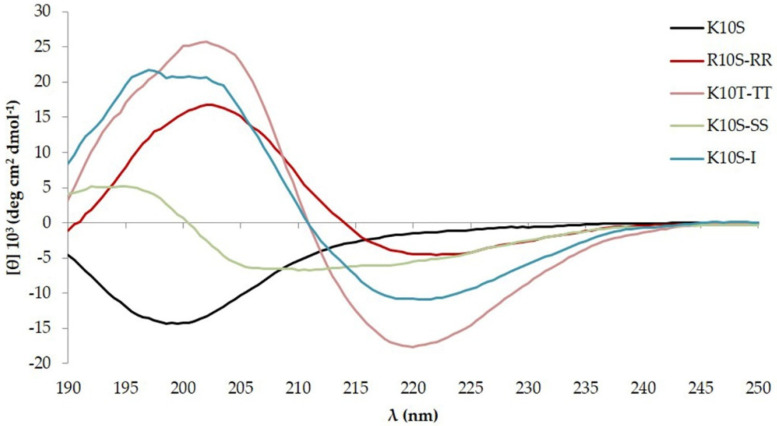
Far UV-CD spectra at 20 °C of 100 μM K10S peptide and its derivatives 2 years after the preparation of the stock aqueous solution (1 mM).

CD spectra of all ISDPs were acquired at time 0, 7 days, and 2 years after the preparation of the starting aqueous solution. CD spectra observed at time 0 and 7 days showed a similar profile for all ISDPs, with a negative band around 198 nm, typical of random coil structures (Figure 2). Different CD spectra were observed after 2 years. While the parent K10S peptide was not able to undergo any transition toward a recognizable organized structure [19], all its derivatives were able to acquire a well-defined secondary structure. In particular, a β-sheet structure was observed for R10S-RR, K10S-TT, and K10S-I, while K10S-SS showed an α-helix conformation (Figure 3).

### 3.5. Apoptosis Induction and ROS Production in C. albicans Cells after Treatment with ISDPs

**Figure 4:**
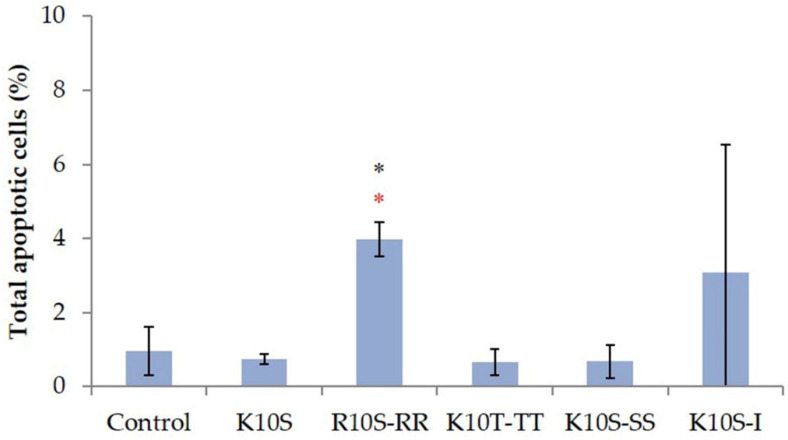
Apoptotic effects of ISDPs in *Candida albicans* SC5314 cells. Phosphatidylserine externalization was analyzed by flow cytometry, after 30 min of treatment with peptides at their double EC_50_ value. Data represent the mean ± standard deviation from at least two independent experiments. The percentage of apoptotic cells after treatment with R10S-RR, although low, was significantly different from that of untreated (control, black *) or parent peptide-treated (red *) cells, as assessed by Student’s *t*-test (*p* < 0.05).

Flow cytometry based on the detection of phosphatidylserine on the surface of yeast cells was used to assess if treatment with ISDPs could induce apoptosis in *C. albicans* SC5314 cells. Under the experimental conditions adopted, only R10S-RR peptide was able to induce apoptosis (*p* < 0.05), although in a low number of cells, unlike the parent K10S peptide (Figure 4).

**Figure 5:**
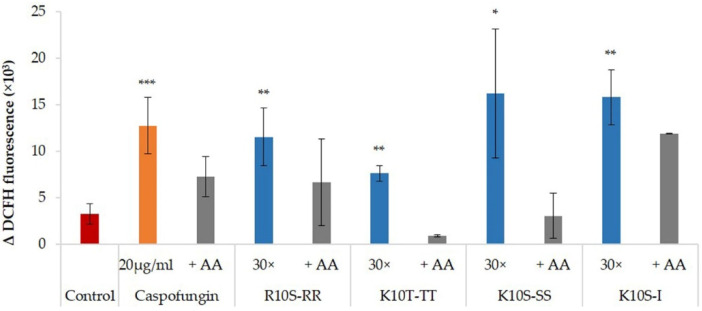
Intracellular reactive oxygen species (ROS) production after incubation of *Candida albicans* SC5314 cells with caspofungin, at 20 µg/mL, and ISDPs, at a concentration equal to 30× EC_50_ value, for 30 min. AA, yeast cells pre-treated with ascorbic acid. Data represent the mean ± standard deviation from at least two independent experiments (* *p* < 0.05, ** *p* < 0.01, *** *p* < 0.001 vs. control untreated cells). Δ fluorescence values (DCFH at 4 h—DCFH at time 0) are shown.

Intracellular ROS production was evaluated in *C. albicans* cells after treatment with ISDPs for 30 min. A green fluorescence resulting from the oxidation of DCFH-DA into DCFH, indicating the presence of ROS, was seen for all ISDPs. As observed with the positive control caspofungin, ROS production was inhibited by the previous treatment with the well-known antioxidant ascorbic acid. In Figure 5, the Δ fluorescence value at 4 h was reported.

### 3.6. Fluorescence Microscopy Studies on C. albicans Cells after Treatment with ISDPs

**Figure 6:**
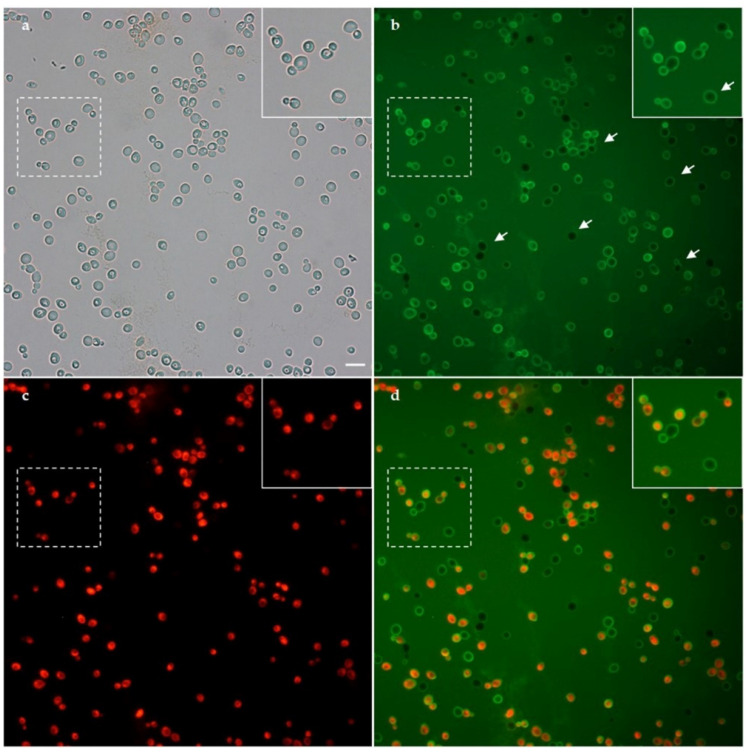
Fluorescence microscopy images of living *Candida albicans* cells pre-treated with lucifer yellow (LY) and propidium iodide (PI), then treated with R10S-RR. (**a**) Light transmission; (**b**) 20 min after the addition of R10S-RR peptide, LY is evidenced inside many yeast cells, suggesting an irreversible peptide-induced membrane permeabilization resulting in cell death, as demonstrated by PI internalization (**c**). Arrows in panels (**b**) indicate LY localization at the cell wall level prior to internalization. The inset shows a detail of the field highlighted by the hatching. (**d**) Merge of (**b**,**c**). Bar, 10 μm.

Fluorescence microscopy allowed to investigate membrane permeabilization in living *C. albicans* cells following ISDPs treatment. An irreversible membrane permeabilization was observed already after 10 min of treatment with R10S-RR and K10S-I, as demonstrated by simultaneous internalization of LY and PI in many yeast cells. In Figure 6, *C. albicans* cells after 20 min treatment with R10S-RR are shown. Instead, membrane permeabilization was very limited in *Candida* cells after 30 min treatment with K10T-TT and K10S-SS peptides.

### 3.7. In Vivo Therapeutic Activity of ISDPs in G. mellonella

**Figure 7:**
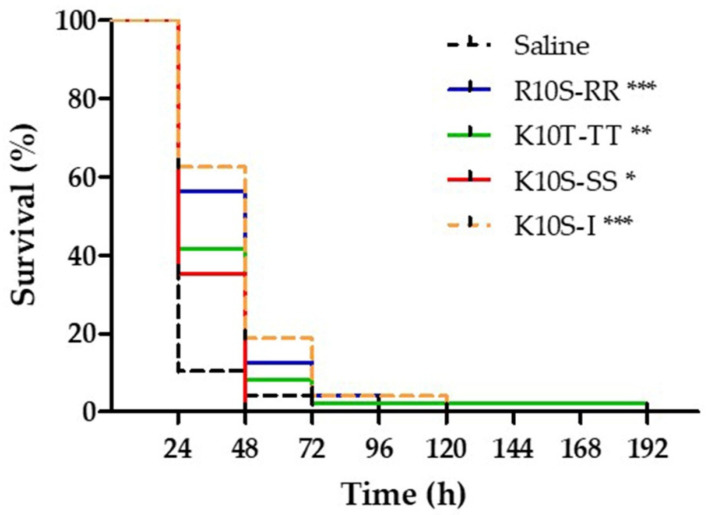
Therapeutic activity of selected peptides. *Galleria mellonella* larvae were infected with 5 × 10^5^ cells of *Candida albicans* SC5314 and treated with peptides (single injection, 13 μmol/kg) or saline solution (control group). The survival curves of all selected peptides were significantly different from that of the control group, as assessed by Mantel–Cox logrank test (* *p* < 0.05, ** *p* < 0.01, *** *p* < 0.001).

Therapeutic activity of ISDPs against *C. albicans* infection was evaluated in *G. mellonella* larvae. After the inoculum of a lethal dose of yeast cells, a single injection of peptides led to a significant increase in survival of larvae in comparison with infected animals inoculated with saline. The median survival time was 48 h for larvae treated with R10S-RR and K10S-I, and 24 h for larvae treated with K10T-TT and K10S-SS, versus 24 h for the control group. In a previous work, a median survival time of 72 h was reported for larvae treated with the parent K10S peptide in comparison with 24 h for controls [25]. Survival curves in Figure 7 report the pooled results obtained in three independent experiments.

## 4. Discussion

The epidemiological relevance of fungal infections requires to expand the limited therapeutic repertoire currently available, with the ultimate goal to reduce the mortality from fungal diseases [25]. In this perspective, peptides of different origin showing antifungal activity, also against drug-resistant strains, are interesting candidate molecules. Recently, a new open-access database (DRAMP) containing over twenty thousand peptides, of which more than three thousand are characterized by antifungal activity, was developed [26]. To overcome the drawbacks of AMPs in relation to their efficacy, in vivo stability, toxicity, and expensive large-scale production, new strategies have been focusing on designing synthetic mimics and developing new delivery systems [15,27,28,29,30]. Moreover, new strategies have been developed for molecular modifications and many peptides have been studied intensively, such as histatin-5 [29], lactoferricins [31], and anoplin [32].

In this contest, in silico studies were performed aiming to identify novel derivatives of the peptide K10S endowed with antifungal activity against *C. albicans*. Amino acid substitutions have been designed in order to understand the role of specific residues in determining the activity of peptides, maintaining a good balance among optimum hydrophobicity, positive charges, hydrogen bonding attitude, and their distribution within the peptide sequence. The results were in agreement with what has already been described by Agrawal et al. [17].

All K10S derivatives showed an increased candidacidal activity in comparison with the parent peptide. In particular, the highest anti-*Candida* activity was observed with Ser/Thr substitutions, confirming the significance of having polar uncharged residues in precise positions within the peptide sequence. Ala replacement with the more hydrophobic Ile amino acid in K10S-I derivative and the double replacement of Lys with Arg residues in R10S-RR peptide at the N-terminus also improved peptide activity in comparison with K10S, demonstrating the importance of maintaining a certain basicity at N-terminus and hydrophobicity at C-terminus. Lys-Arg substitution has already been reported for lactoferricin [33]. It is now well established that an increase in hydrophobicity and amphipathicity usually correlates with an increase in antifungal activity. These features are crucial for peptide–membrane interactions and membrane permeabilization [34] and are important variables to consider for the design of synthetic peptides.

All the results obtained with the ISDPs derived from K10S allow to suggest a consensus sequence endowed by basic residues at the N-terminus and two hydrophobic aminoacid spaced by five residues characterized by Ser or Thr alternating with generic amino acids (Basic-Hyd-T/S-X-T/S-X-T/S-Hyd).

Enough time for peptides to find the best possible conformation from an energetic point of view was offered by performing CD analysis 2 years after the preparation of the stock aqueous solution. Acquired CD spectra showed well-defined secondary structures for the investigated derivatives, unlike the parent peptide. This diversity could be traced back to specific characteristics of the substituted amino acids. In particular, Arg residues in R10S-RR peptide are involved in a greater number of hydrogen bonds than Lys in K10S parent peptide, favoring the secondary structure acquisition; in the same way, Ala substitution with a more hydrophobic amino acid (Ile) in K10S-I peptide favored inter-strand hydrophobic interactions. Thr residues introduced the propensity for the formation of β-sheet structure [35], while Ser substitutions owing to minor bulkiness favored the α-helical conformation. The activity of K10T-TT and K10S-SS correlates to the GRAVY parameter, which considers the hydrophobicity of amino acid residues. However, the structural differences between peptides do not seem to correlate with antifungal activity; indeed, the two peptides with the highest anti-*Candida* activity, K10S-TT and K10S-SS, are structured in β-sheet and α-helix, respectively.

The individual amino acid substitutions also influenced the possible mechanism of action of the peptides. For the parent peptide K10S, a non-membranolytic mechanism of action has been hypothesized, with the induction of intracellular ROS production, a rapid decrease in mitochondrial transmembrane potential, and the lack of induction of apoptotic processes [19]. All ISDPs were characterized by the ability to induce intracellular ROS production (Figure 5). K10T-TT and K10S-SS showed a behavior similar to the parent peptide, although with faster killing kinetics, which could explain their lower EC_50_ value. Instead, for R10S-RR and K10S-I, a peptide-induced membrane permeabilization has been assumed. In particular, R10S-RR was characterized by a very fast killing kinetics; indeed, after 5 min of treatment with this ISDP more than 70% cells were dead (Figure 1). This behavior is compatible with a membranolytic mechanism of action, also confirmed by studies in fluorescence microscopy (Figure 6). After a few minutes of peptide treatment, many yeast cells showed a simultaneous internalization of LY and PI. The reasons for this change from the parent peptide could be mainly due to physico-chemical characteristics of the Arg residue, positively charged at physiological pH as Lys, but with a lower hydropathicity value [36], a critical feature for the interaction of the peptide with the target microbial cells. The ability to induce irreversible membrane permeabilization has also been hypothesized for K10S-I, in relation to the greater hydrophobicity of this peptide. A unique aspect of the R10S-RR derivative in comparison with the parent K10S and other ISDPs was its ability to induce apoptosis under experimental conditions, though in a low percentage of treated yeast cells.

The possible hemolytic and cytotoxic effects of all ISDPs were also investigated, excluding damage against human red blood and LLC-MK2 cells, respectively. Last, but not least, the potential interest of the investigated peptides was confirmed by the observation that all ISDPs showed a therapeutic effect in vivo in the experimental systemic candidiasis model in *G. mellonella* larvae.

In conclusion, the in silico design of K10S derivatives followed by their synthesis has proven to be an effective strategy in producing novel antifungal peptides and in determining a rather precise consensus (Basic-Hyd-T/S-X-T/S-X-T/S-Hyd) for the identification and/or development of new peptides endowed with anti-*Candida* activity. As a future perspective, we plan to characterize further substitutions of the K10S peptide and to apply our approach to the optimization of D5A and N1A peptides.

## Figures and Tables

**Table 1:** Sequence alignment of selected antimicrobial peptides.

Peptide	Sequence Alignment
K10S	**-KKVTMTCSAS**
D5A	**TCRVAHRGLTF**
N1A	**-AQVSLTCLVK**

Red: identical residue in the three sequences; blue: identical residue in two sequences; green: residue with similar chemical-physical characteristics.

**Table 2:** Amino acid sequences and characteristics of the parent peptide and the selected derivatives.

Peptide	Sequence	Substitution	M.M. (Da)	GRAVY Value	pI	Net Charge
K10S (parent)	KKVTMTCSAS	-	1055.27	−0.040	9.31	2+
R10S-RR	RRVTMTCSAS	Basic	1111.30	−0.160	10.35	2+
K10T-TT	KKVTMTCTAT	Ser/Thr	1083.33	−0.020	9.31	2+
K10S-SS	KKVSMSCSAS	Thr/Ser	1027.22	−0.060	9.31	2+
K10S-I	KKVTMTCSIS	Hydrophobic	1097.35	0.230	9.31	2+

M.M. (Da), molecular mass (Dalton); GRAVY, grand average of hydropathy; pI, isoelectric point. In red, substituted residue; M.M., GRAVY, pI, and charge calculated by ExPASy tool ProtParam.

**Table 3:** In vitro candidacidal activity of in silico designed peptides (ISDPs) against *C. albicans* SC5314.

Peptide	EC_50_^1^ (95% Confidence Intervals)	EC_50_ ISDP/EC_50_ K10S
K10S	0.277 (0.274–0.279) ^2^	-
R10S-RR	0.254 (0.247–0.262)	0.92
K10T-TT	0.173 (0.171–0.175)	0.62
K10S-SS	0.159 (0.150–0.168)	0.57
K10S-I	0.260 (0.239–0.282)	0.94

^1^ EC_50_, half maximal effective concentration, [mol/liter] × 10^−6^; ^2^ EC_50_ of K10S parent peptide [19].

## Data Availability

The data presented in this study are available in the article.
